# Epidemiological features of acute medial meniscus posterior root tears

**DOI:** 10.1007/s00264-023-05848-0

**Published:** 2023-06-17

**Authors:** Yusuke Kamatsuki, Takayuki Furumatsu, Takaaki Hiranaka, Yuki Okazaki, Keisuke Kintaka, Yuya Kodama, Shinichi Miyazawa, Toshifumi Ozaki

**Affiliations:** 1 Department of Orthopaedic Surgery, Okayama University Hospital, 2-5-1 Shikatacho, Kitaku, Okayama 700-8558 Japan; 2 Department of Orthopaedic Surgery, Kochi Health Sciences Center, 2125-1 Ike, Kochi, 781-8555 Japan

**Keywords:** Body mass index, Medial meniscus, Painful popping, Posterior root tear, Pullout repair

## Abstract

**Purpose:**

Untreated or overlooked medial meniscus posterior root tears (MMPRTs) induce sequential knee joint degradation. We evaluated epidemiological features of acute MMPRT for its early detection and accurate diagnosis.

**Methods:**

Among 330 MMPRT patients from 2018 to 2020, those who underwent arthroscopic pullout repairs were enrolled. Patients who underwent non-operative treatment or knee arthroplasty, those with a cruciate ligament-deficient knee or advanced osteoarthritis of the knee, and those with insufficient data were excluded. Finally, we retrospectively evaluated data from 234 MMPRTs (female: 79.9%, complete tears: 92.7%, mean age: 65 years). Welch’s t-test and Chi-squared test were used for pairwise comparisons. Spearman’s rank correlation analysis was performed between age at surgery and body mass index (BMI). Multivariable logistic regression analysis with stepwise backward elimination was applied to the values as risk factors for painful popping events.

**Results:**

In both sexes, there were significant differences in height, weight, and BMI. In all patients, there was a significant negative correlation between BMI and age (*ρ* =  − 0.36, *p <* 0.001). The BMI cutoff value of 27.7 kg/m^2^ had a 79.2% sensitivity and a 76.9% specificity for detecting MMPRT patients aged < 50 years. A painful popping event was confirmed in 187 knees (79.9%), and the frequency was significantly reduced in partial tears as compared to complete tears (odds ratio: 0.080, *p <* 0.001).

**Conclusion:**

Higher BMI was associated with a significantly younger age of MMPRT onset. Partial MMPRTs had a low frequency of painful popping events (43.8%).

## Introduction

Medial meniscus (MM) posterior root tears (MMPRTs), which mainly occur in women aged over 50 years, are usually heralded by painful popping during light activity, such as descending stairs or walking [1–3]. MMPRT frequency was reportedly 21.5% in all arthroscopic surgeries for MM tears [4]. Untreated or overlooked MMPRTs induce sequential knee joint degradation. Long-term follow-up studies revealed that MMPRT pullout repair was an effective intervention to protect the knee joint in terms of clinical outcomes and survivorship [5, 6]. Therefore, the favourable treatment of MMPRT is an arthroscopic repair for cases with mild or no osteoarthritic changes [7, 8]. However, patients with progressed knee osteoarthritis (OA) or spontaneous osteonecrosis of the knee, which is considered a subchondral insufficiency fracture of the knee (SIFK) associated with chronic MMPRTs, are commonly observed [9, 10]. Among 197 knees that underwent total knee arthroplasty for OA, 78.2% suffered MMPRT [10]. Confirmation of a painful popping event is useful for MMPRT diagnosis with high specificity [2, 3]. Without a clear popping event, it is difficult to diagnose an MMPRT and determine whether magnetic resonance imaging (MRI) should be performed [2].

Among several risk factors of MMPRT, such as older age, female sex, higher body mass index (BMI), increased Kellgren–Lawrence (K–L) grade, and a steep posterior slope of the medial tibial plateau [4, 11], BMI is one of the few modifiable risk factors. In a recent study, BMI ≥ 25 kg/m^2^ was reportedly associated with OA progression in patients who underwent conservative MMPRT treatment [12]. Furthermore, an elevated BMI was associated with worse clinical outcomes following MMPRT [13, 14]. Hence, it was hypothesised that BMI would also affect the timing of MMPRT onset. We aimed to determine whether sex, BMI, and type of tear were associated with MMPRT onset and injury pattern.

## Materials and methods

### Ethical approval and study design

This study was approved by the Institutional Review Board of our institution and conducted according to the Declaration of Helsinki. All patients provided written informed consent before participation. Data of patients who visited our hospital and were diagnosed with MMPRTs from January 2018 to November 2020 were retrospectively collected. Patients who underwent non-operative treatment or total/unicompartmental knee arthroplasty were excluded because related MMPRT onset was mostly chronic or unclear.

All patients who underwent an arthroscopic pullout repair were included. Inclusion criteria were arthroscopic pullout repair of the MMPRT performed for patients with a femorotibial angle (FTA) ≤ 180°, a K–L grade of 0–2, and a mild cartilage lesion (modified Outerbridge grade I or II) that was confirmed by preoperative radiographs and MRI examinations [15, 16]. MMPRT patients underwent arthroscopic pullout repair as previously described [17–19]. Exclusion criteria comprised patients with an anterior cruciate ligament (ACL)- or posterior cruciate ligament (PCL)-deficient knee and those with insufficient preoperative radiographic data.

Age at surgery, sex, height, weight, BMI, and the duration from injury to arthroscopic pullout repair were recorded for each patient. Details of posteromedial painful popping episodes (a predictive sign of MMPRTs in middle-aged to older patients [2]), including the injury situation, position of the injured leg, and injury date, were obtained from patients via careful interviews at the first visit. Painful popping was defined as a clear single acute onset of severe pain around the posteromedial knee. Patients were usually unable to walk on the injured leg due to severe pain immediately after the popping event, occasionally accompanied by cold sweats. Absence of painful popping was considered in patients who had no painful popping events but clearly remembered that they started to feel posteromedial knee pain during activities or after traumatic injuries, such as falls. If patients could not clearly recall their painful popping event, they were defined as uncertain. The operation records were reviewed to determine the posterior root tear classification and ACL/PCL condition.

Preoperative knee deformity was assessed using coronal radiological FTA and K–L arthritis grade. FTA was defined as the external angle between the femoral and tibial shaft axes on coronal radiographs of the entire lower limbs in the standing position. K–L grades on the posteroanterior 45°-flexion Rosenberg standing view and spontaneous osteonecrosis of the knee stages on the anteroposterior (AP) view were determined according to the classifications [16, 20].

### MMPRT evaluation

MMPRT type was identified according to the classification of tear morphology as follows: type 1, partial stable meniscal tears within 9 mm of the centre of the root attachment; type 2, complete radial tears within 9 mm of the centre of the root attachment; type 3, bucket-handle tears with meniscal root detachment; type 4, complex oblique meniscal tears extending into the root attachment; and type 5, avulsion fractures of the meniscal root attachment [21]. Partial and complete tear groups included patients with type 1 and type 2–4 tears, respectively.

### Statistical analyses

Data were expressed as mean ± standard deviation (SD) unless indicated otherwise. Statistical significance was set at *p <* 0.05. Welch’s t-test was used to compare data of the female and male groups. Chi-squared test was used to compare sex distribution, MMPRT type, and K–L grade between two groups with partial and complete tears. Spearman’s rank correlation analysis was performed between age at surgery and BMI. A multivariable logistic regression analysis with stepwise backward elimination was applied to the values as risk factors for painful popping events. Statistical calculations and the receiver operating curve (ROC) construction were performed using EZR-WIN software (Saitama Medical Center, Saitama, Japan). The optimal BMI cutoff associated with the age of MMPRT onset was determined using ROC and the Youden index (J). Power analysis was performed using G*Power version 3.1 (Heinrich Heine University, Düsseldorf, Germany). The sample size was estimated for a minimal statistical power of 95% (α = 0.05). In the Spearman’s rank correlation analysis, a sample of 134 knees was considered sufficient to detect an effect size of d = 0.30 with 95% statistical power.

## Results

**Fig. 1:**
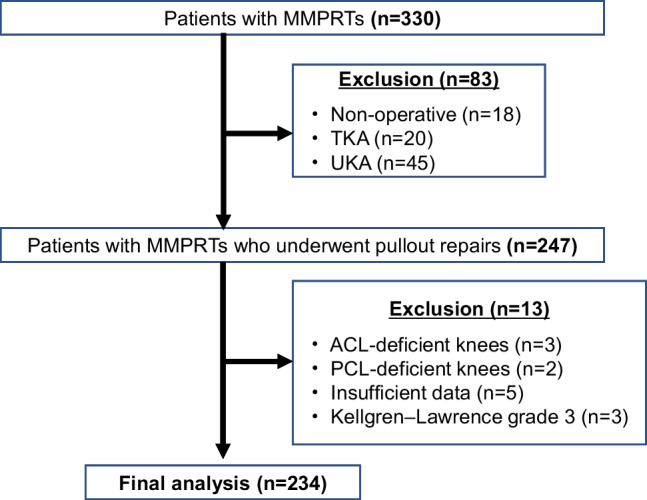
Flowchart of the study protocol. MMPRT, medial meniscus posterior root tear; TKA, total knee arthroplasty; UKA, unicompartmental knee arthroplasty; ACL, anterior cruciate ligament; PCL, posterior cruciate ligament

**Fig. 2:**
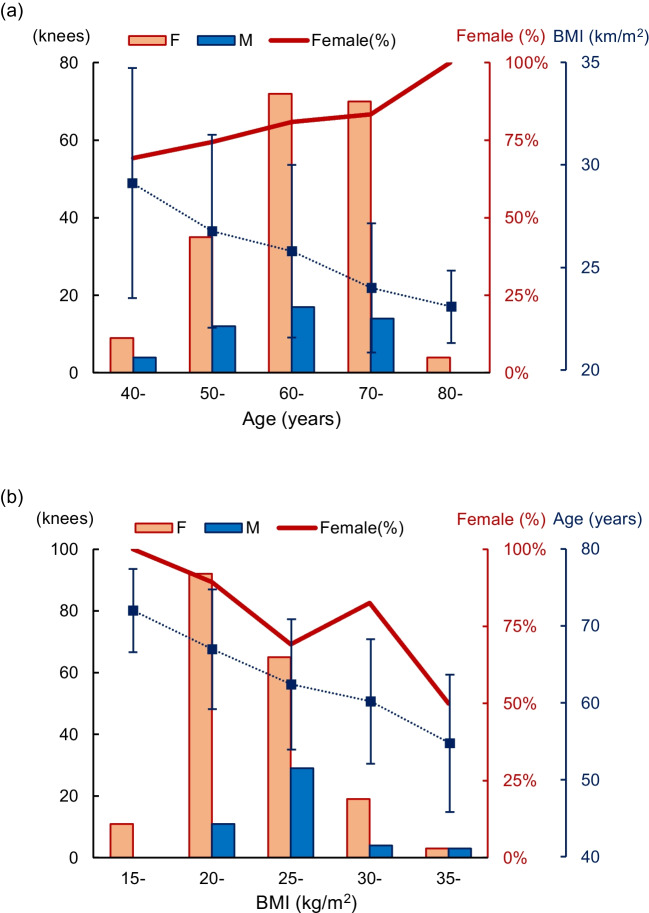
Patient distribution by age and body mass index (BMI). Black squares and bars represented (**a**) mean BMI ± standard deviation (SD) for several ages and (**b**) mean age ± SD for several BMIs

In the final analysis, 234 MMPRTs in 231 patients were included (Fig. 1). All patients were Lachman test-negative. No patient had a concomitant collateral ligament injury in the affected knee. Patient demographics are shown in Table 1. Female patients accounted for 79.9% of the total number of patients. There were significant differences between male and female patients in height, weight, and BMI (Table 2). The proportion of female patients increased as the patients’ age increased (Fig. 2a). The peak proportion for BMI was higher for male than for female patients (Fig. 2b). The proportion of female patients and the mean age of the patients decreased as BMI increased.

**Table 1:** Patient demographics (*n =* 234)

		Range
Sex (male/female, knees)	47/187	
Affected side (right/left)	109/125	
Age (years)	64.9 (8.5)	41–86
Height (m)	1.57 (0.08)	1.32–1.92
Weight (kg)	63.1 (13.5)	40–120
Body mass index (kg/m^2^)	25.6 (4.2)	17.0–45.9
Duration from injury to operation (days)^#^	68 (51)	9–310
Popping event ( +/-/unclear)	187/29/(18)	
MMPRT type (1/2/3/4/5)	17/192/1/24/0	
SONK stage (none/1/2/3/4)	223/9/1/1/0	
Kellgren-Lawrence grade (0/1/2/3/4)	0/85/149/0/0	
Femorotibial angle (°)	177.3 (1.6)	172–180
Posterior tibial slope (°)	9.0 (2.6)	2–15

Data are presented as number of cases or mean (standard deviation)

MMPRT, medial meniscus posterior root tear; SONK, spontaneous osteonecrosis of the knee

^#^ excluding 18 patients without a clear memory of painful popping

**Table 2:** Comparison between male and female

	Male (*n =* 47)	Female (*n =* 187)	*p*
Affected side (right/left)	18/29	91/96	0.20
Age (years)	63.0 (9.3)	65.4 (8.2)	0.12
Height (m)	1.68 (0.07)	1.54 (0.06)	** < 0.001**
Weight (kg)	78 (14)	59 (11)	** < 0.001**
Body mass index (kg/m^2^)	27.5 (4.0)	25.1 (4.1)	**0.001**
Duration from injury to operation (days)^#^	69 (52)	68 (52)	0.86
Popping event ( +/-/unclear)	37/6/(4)	150/23/(14)	0.97
MMPRT type (1/2/3/4)	1/41/1/4	16/151/0/20	0.09
SONK stage (none/1/2/3)	46/0/1/0	177/9/0/1	0.09
Kellgren-Lawrence grade (1/2)	19/28	66/121	0.51
Femorotibial angle (°)	177.3 (1.5)	177.3 (1.7)	0.98
Posterior tibial slope (°)	9.3 (2.6)	9.0 (2.6)	0.47

Bold indicates *p* < 0.05

Data are presented as number of cases or mean (standard deviation)

MMPRT, medial meniscus posterior root tear; SONK, spontaneous osteonecrosis of the knee

^#^excluding 18 patients without a clear memory of painful popping

**Fig. 3:**
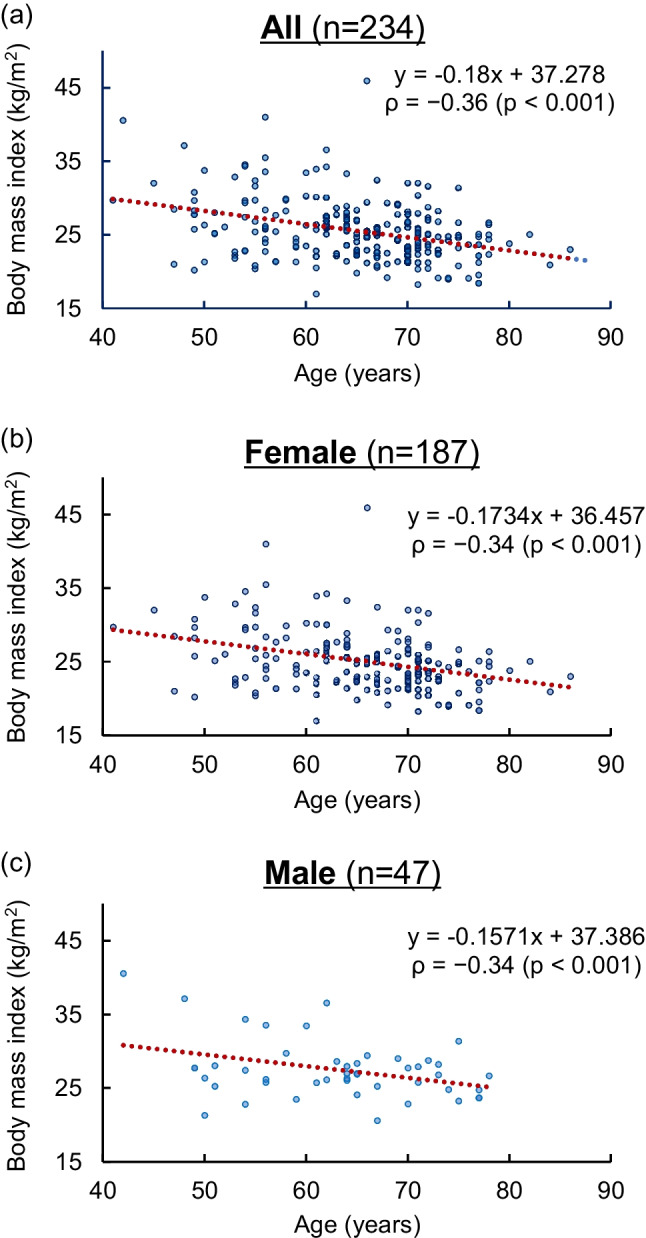
Correlation analysis of body mass index and age. (**a**) All knees. *ρ =*  − 0.36, *p <* 0.001*.* (**b**) Female knees. *ρ =*  − 0.34, *p <* 0.001. (**c**) Male knees. *ρ =*  − 0.34, *p* < 0.001. *ρ*, Spearman’s rank correlation coefficient

**Fig. 4:**
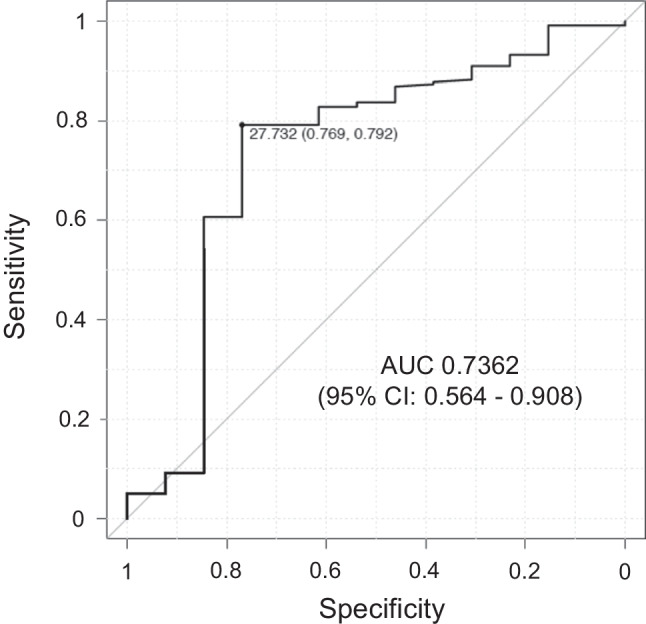
The optimal cutoff value for body mass index (BMI) to detect MMPRT patients under 50 years of age. The BMI cutoff value of 27.7 kg/m^2^ had a 79.2% sensitivity and a 76.9% specificity for MMPRT detection in patients under 50 years of age. AUC, area under the curve; CI, confidence interval; MMPRT, medial meniscus posterior root tear

In correlation analysis, there was a significant negative association between BMI and age in all patients as well as the female and male groups (*ρ =*  − 0.36 to − 0.34, *p <* 0.001; Fig. 3). In 47.0% (110/234) of the knees, the corresponding BMI was < 25 kg/m^2^, suggesting that these patients had normal weight or were underweight based on the World Health Organisation criteria. ROC analysis identified the optimal BMI cutoff value of 27.7 kg/m^2^ for MMPRT detection in younger patients aged < 50 years (sensitivity: 79.2%, specificity: 76.9%; Fig. 4).

**Fig. 5:**
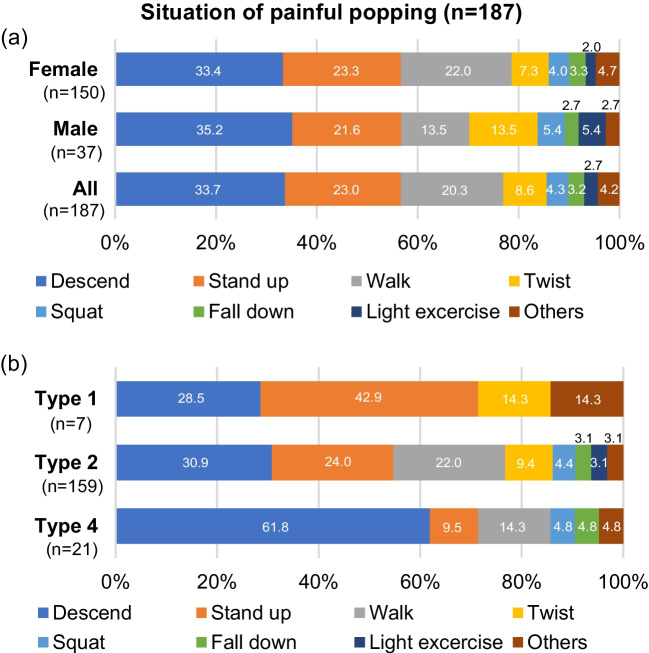
Distribution of injury patterns by (**a**) sex and (**b)** type of medial meniscus posterior root tear

Type 2 was the most frequent MMPRT type (82.0%), followed by type 4 (10.3%), type 1 (7.3%), and type 3 (0.4%) (Table 1). A clear painful popping event was confirmed in 187 out of 234 knees (79.9%). Painful popping mainly occurred when patients stepped during the knee motion that was involved in descending (33.7%), standing up (23.0%), and walking (20.3%) actions (Fig. 5a). In MMPRT type 4, descending motion had a significantly higher rate of MMPRT injury than that of MMPRT type 2 (13/21 knees [61.8%] vs. 49/159 knees [30.9%], *p* = 0.004; Fig. 5b).

Univariate analysis of the association between the presence of a painful popping event and clinical factors revealed a significant difference between affected sides (left vs. right: 82.2% vs. 91.8%, *p* = 0.04) and between partial and complete tears (43.8% vs. 90.0%, *p <* 0.001; Table 3). The logistic regression model indicated that the odds of a painful popping event decreased with a partial tear (odds ratio [OR]: 0.07, 95% confidence interval [CI] 0.02–0.24, *p <* 0.001; Table 3).

**Table 3:** Univariate analysis and logistic regression analysis; association between the presence of popping event and clinical factors

	Popping (+) (*n* = 187)	Popping (-) (*n* = 29)	*p*-value	Odds ratio (95% CI)	*p*-value
Age (years)					
< 60	49	6	0.53		
60 ≤	138	23			
Sex					
Female	150	23	0.91		
Male	37	6			
Affected side					
Left	97	21	**0.04**	1 (reference)	
Right	90	8		2.28 (0.89–5.84)	0.08
Body mass index (kg/m^2^)					
< 25	88	13	0.20	1 (reference)	
25 ≤, < 30	73	15		0.69 (0.28–1.67)	0.41
30≤	26	1		4.17 (0.46–37.6)	0.20
MMPRT type					
Partial tear (type 1)	7	9	**<0.001**	**0.07** (0.02–0.24)	**<0.001**
Complete tear (type2/3/4)	180 (159/0/21)	20 (16/1/3)		1 (reference)	
Kellgren-Lawrence grade					
1	70	10	0.76		
2	117	19			
Femorotibial angle (°)					
≤ 177	92	17	0.35		
177 <	95	12			
Posterior tibial slope (**°**)					
≤ 9	105	12	0.13	1 (reference)	
9 <	82	17		0.54 (0.23–1.31)	0.14

Bold indicates p < 0.05. MMPRT, medial meniscus posterior root tear

## Discussion

This study demonstrated that a higher BMI was significantly associated with a younger age of MMPRT onset, and a significant difference in injury pattern was observed for MMPRT type 4, which mainly occurred due to descending motion, as compared with other MMPRT types. Furthermore, the frequency of painful popping events was significantly reduced in partial MMPRTs (type 1) than in complete tears (types 2–4). Moreover, the proportion of female patients increased with increasing patient age, and the proportion of male patients increased with increasing BMI. In clinical practice, these findings would be useful for early and reliable detection of MMPRTs.

The associations among higher BMI, younger MMPRT onset, and female patients (Figs. 2 and 3) are consistent with the distribution of the general population with knee OA, which could result from MMPRT, a contributing cause of knee OA. To our best knowledge, this is the first study to demonstrate a relationship between BMI and the age of MMPRT onset, which could explain the increasing proportion of male patients with younger ages and higher BMI values (Fig. 2).

The meniscotibial ligament is associated with meniscal extrusion and MMPRT, and abnormality or disruption of the meniscotibial ligament might have already been present before MMPRT onset [22, 23]. In an ultrasound-based study, increasing age, BMI, and load were significantly correlated to increase meniscus extrusion [24]. Therefore, in addition to the synergistic effect of age and BMI on MMPRT onset, high BMI (> 30 kg/m^2^) itself could accelerate MMPRT onset. In this study, BMI ≥ 27.7 kg/m^2^ was the optimal cutoff value to detect the younger age of MMPRT onset (Fig. 4). This finding was consistent with the demographic data of patients from two previous long-term follow-up studies, in which Bernard et al. [5] included younger and heavier patients than Chung et al. [6] (mean age: < 49 years vs. > 56 years, mean BMI: > 32 kg/m^2^ vs. < 28 kg/m^2^). Moreover, these findings indicated that older patients with no or mild knee OA could develop MMPRT despite not being obese. This study included 58 knees from patients aged > 70 years with a BMI < 25 kg/m^2^. Orthopaedic surgeons should carefully identify atypical patients, such as those who are older and not obese.

In our study, female patients accounted for 79.9% of all MMPRT patients in the final analysis. This female proportion was similar to those from other studies, with female participants representing 64.0–90.9% of all MMPRT patients [5, 6, 13, 25, 26]. An explanation for this phenomenon remains to be determined. Sex differences may play a role in the development of MMPRTs due to differences in knee joint bone geometry. Studies have identified steep medial posterior tibial slope (PTS) and narrow intercondylar notch width as potential risk factors for MMPRT [27, 28]. Additionally, research has shown that females generally have a steeper medial PTS and smaller intercondylar notch width than males, which could contribute to their higher risk of developing MMPRT [29–31]. Furthermore, female patients generally have more joint laxity and less muscle strength than male patients [32, 33]. Sex-related hormonal differences may also influence gender differences in the occurrence of MMPRT. Several studies have identified differences in knee laxity across the female menstrual cycle and pregnancy [34–36]. Incidence rates of OA in men and women diverge around the age of 50 years, corresponding to the onset of menopause and the associated decline in systemic oestrogen levels in women [37], which may be closely linked to the high incidence of MMPRT among females. Therefore, sex differences in the morphological, biomechanical, and biological aspects described above may contribute to the higher incidence of MMPRT in females than in males. However, in the present study, there were no significant differences in PTS between both sexes (Table 2), and intercondylar notch width and hormones were not evaluated. Further studies are warranted to investigate the association between these factors and the high frequency of MMPRT in women.

In 187 knees with a painful popping event, descending motion (33.7%) was the most frequently detected injury pattern, which was consistent with the findings of a previous study [3], followed by activities of daily living, such as standing up (23.0%) and walking (20.3%). Additionally, activities of daily living, including twisting (8.6%) and squatting (4.3%), accounted for 89.9% of all injury patterns with a clear painful popping event. In a biomechanical study, the MM posterior horn or root mainly carried the posterior shear load, particularly after 30° knee flexion [38]. In a simulated-gait analysis using cadaveric knees, the MM posterior aspect distributed the peak load during the early phase of stance [39]. Therefore, the MM posterior root, functioning as a dike-like secondary stabiliser of the knee, could have been regularly and repeatedly exposed to load during activities of daily living until a relatively minor load during stepping on stairs and walking triggered the MMPRT onset. This potential mechanism of injury could explain for the distribution of injury patterns observed in our study. However, the cause of a significantly higher injury rate during descending motion in MMPRT type 4 as compared to another type of complete tear (type 2) remains unclear. Rearfoot landing in stairs or ramps while descending reportedly increased knee joint loads to a greater degree as compared to forefoot landing for patients with early knee OA [40], and further research of this phenomenon is required.

In this study, partial MMPRTs had a significantly reduced frequency of painful popping events as compared with complete tears, which could explain for the low sensitivity and, conversely, high specificity of painful popping events for MMPRT detection [2]. This finding also suggested that it may be difficult for some MMPRT patients to recognise when they have injured their knees because of their less-acute subjective symptoms, which can result in more overlooked or unrecognised MMPRT patients than expected. Therefore, it is important that orthopaedic surgeons consider the possibility of MMPRT, particularly type 1, and the necessity of MRI in patients with continuous knee pain or effusion who are over 40 years of age, including those who do not report or do not remember experiencing a painful popping event.

This study had few limitations. First, only patients who underwent arthroscopic pullout repair for MMPRT were included, which might impose a selection bias. Therefore, it might be inappropriate to apply these findings to all MMPRT patients, including patients receiving non-operative treatment, those requiring arthroplasty for progressed OA, or those with SIFK. However, we included patients with acute MMPRT and mild knee OA because the mean duration from injury to arthroscopic pullout repair was 68 days, and 187 out of 234 patients (79.9%) had a clear single painful popping event. Additionally, since our surgical indications are similar to those of other institutions [41, 42], we believe our study results would, at least, reflect the epidemiological features of acute MMPRT. Second, this study did not include a control group. Third, since this study included a single ethnic group, whether our findings are applicable to other races or individuals with different lifestyles remains unclear. Fourth, we did not evaluate the degree of meniscal extrusion and its correlation with clinical symptoms. Nevertheless, this study is clinically relevant because it revealed variables associated with patients with MMPRT diagnosis in more detail as compared to previous studies.

## Conclusion

Higher BMI was associated with a significantly younger age of MMPRT onset. The likelihood of a painful popping event was significantly lower in partial MMPRTs than in complete tears. Understanding these features will be useful for early detection of MMPRTs.

## Data Availability

The data are not available due to their containing information that could compromise the privacy of research participants.
